# Root pathogen diversity and composition varies with climate in undisturbed grasslands, but less so in anthropogenically disturbed grasslands

**DOI:** 10.1038/s41396-020-00783-z

**Published:** 2020-09-21

**Authors:** Camille S. Delavaux, Josh L. Schemanski, Geoffrey L. House, Alice G. Tipton, Benjamin Sikes, James D. Bever

**Affiliations:** 1 Department of Ecology and Evolutionary Biology, The University of Kansas, 2041 Haworth Hall, 1200 Sunnyside Avenue, Lawrence, KS 66045 USA; 2 Kansas Biological Survey, The University of Kansas, 106 Higuchi Hall, 2101 Constant Ave, Lawrence, KS 66047 USA; 3 National Ecological Observatory Network, Boulder, CO 80301 USA; 4 Department of Science, Technology, and Mathematics, Lincoln University, 821 Taylor Drive, 205 Daniel Hall, Jefferson City, MO 65101 USA

**Keywords:** Fungal ecology, Soil microbiology, Grassland ecology

## Abstract

Soil-borne pathogens structure plant communities, shaping their diversity, and through these effects may mediate plant responses to climate change and disturbance. Little is known, however, about the environmental determinants of plant pathogen communities. Therefore, we explored the impact of climate gradients and anthropogenic disturbance on root-associated pathogens in grasslands. We examined the community structure of two pathogenic groups—fungal pathogens and oomycetes—in undisturbed and anthropogenically disturbed grasslands across a natural precipitation and temperature gradient in the Midwestern USA. In undisturbed grasslands, precipitation and temperature gradients were important predictors of pathogen community richness and composition. Oomycete richness increased with precipitation, while fungal pathogen richness depended on an interaction of precipitation and temperature, with precipitation increasing richness most with higher temperatures. Disturbance altered plant pathogen composition and precipitation and temperature had a reduced effect on pathogen richness and composition in disturbed grasslands. Because pathogens can mediate plant community diversity and structure, the sensitivity of pathogens to disturbance and climate suggests that degradation of the pathogen community may mediate loss, or limit restoration of, native plant diversity in disturbed grasslands, and may modify plant community response to climate change.

## Introduction

Experimental and theoretical evidence show that plant pathogens play an important role in structuring plant communities, especially in maintaining plant community diversity [1–3]. For example, soil pathogen accumulation near mature trees is a likely driver of poor performance by seedlings of the same species [4–8]. This pathogen suppression of conspecific seedlings can give heterospecific species the opportunity to succeed in these patches, resulting in a more diverse plant community. Soil pathogens are a major cause for the negative feedback commonly observed between plants and their soil communities, a mechanism which maintains large-scale patterns of plant diversity [9–11]. Similarly, when plants move out of their native range, release from pathogens may help drive their successful invasion of new regions [12, 13], further evidence for the critical role pathogens play in structuring plant communities. Given the important role of pathogens in plant community structure and diversity, responses of plant communities to perturbations may be mediated by the sensitivities of their pathogen communities.

Fungi and fungus-like organisms are major soil-borne plant pathogens. While we know how fungi generally respond to both edaphic properties [14–17] and climate [18–24], it is unclear if plant pathogens mirror these broader responses to environmental factors. Individual studies have shown that pathogen composition in root-infecting fungi is driven primarily by soil pH [17], while diversity has been shown to respond to precipitation [25] and richness to respond to vapor pressure deficit [24]. The composition of oomycetes, common, fungus-like pathogens, depends on a combination of edaphic traits and environmental conditions, including soil pH, nitrogen, phosphorus, latitude, air temperature, and precipitation [23, 26, 27]. Water availability is likely to be particularly important for oomycete distribution, as wet conditions are required for most oomycete zoospore release and flagellar movement [28]. Plant pathogens are also likely to be affected by anthropogenic disturbance, given the large effect of disturbance on plant communities. Land use disturbances such as tillage, fertilizer additions, heavy grazing, and row crop monocultures have been shown to alter mycorrhizal fungal communities [29, 30], but the sensitivity of plant pathogens to disturbance are not well understood, particularly if their responses interact with climate. Understanding plant pathogen responses to environmental drivers is particular important given contemporary and future pressure from anthropogenic change, including changes in land use, temperature, and the intensity and frequency of precipitation events [31, 32]. Because root-associated plant pathogens have large effects on their plant hosts, their responses to climate and land use may mediate plant responses to these anthropogenic impacts.

Here, we use a naturally occurring climate gradient across United States grasslands to investigate root-associated plant pathogen response to climate gradients and anthropogenic disturbance. Specifically, we compare root-associated plant pathogen community diversity and composition across remnant, native grasslands (those without anthropogenic disturbances), and disturbed grasslands (those with a history of anthropogenic disturbances) across a Midwestern US precipitation and temperature gradient from Illinois to Oklahoma. We focus on two groups of root-associated pathogens: fungal pathogens and oomycetes. We hypothesize that root-associated pathogen community structure will be strongly impacted by anthropogenic disturbance, and drive differences in community responses to climate. Undisturbed native grasslands should show the greatest sensitivities to climate variables, such as precipitation and temperature, because the long co-evolutionary history of plants and pathogens there should allow differentiation with respect to climate. Disturbed grasslands are likely dominated by fewer pathogen species, many of which are disturbance-adapted and therefore less sensitive to climate variables. In addition, these disturbed sites likely harbor more homogenous plant communities to serve as pathogen hosts, possibly acting as a filter for establishment of plant pathogens and reducing the range of pathogen response to climate across these sites. Both temperature and precipitation should limit pathogen diversity in undisturbed, native grasslands [33], such that increasing precipitation or temperature will increase pathogen diversity and shift community composition. Precipitation and temperature effects may interact, as has been shown for bacteria [34], overall microbial communities [33], and soil respiration [35]. Finally, because fungal pathogens are phylogenetically distributed throughout the fungal kingdom, we compare fungal pathogen results to those of fungal saprotrophs (decomposers) to determine if responses are pathogen-specific or in line with broader variation in the fungal community.

## Materials and methods

### Field sampling

**Fig. 1:**
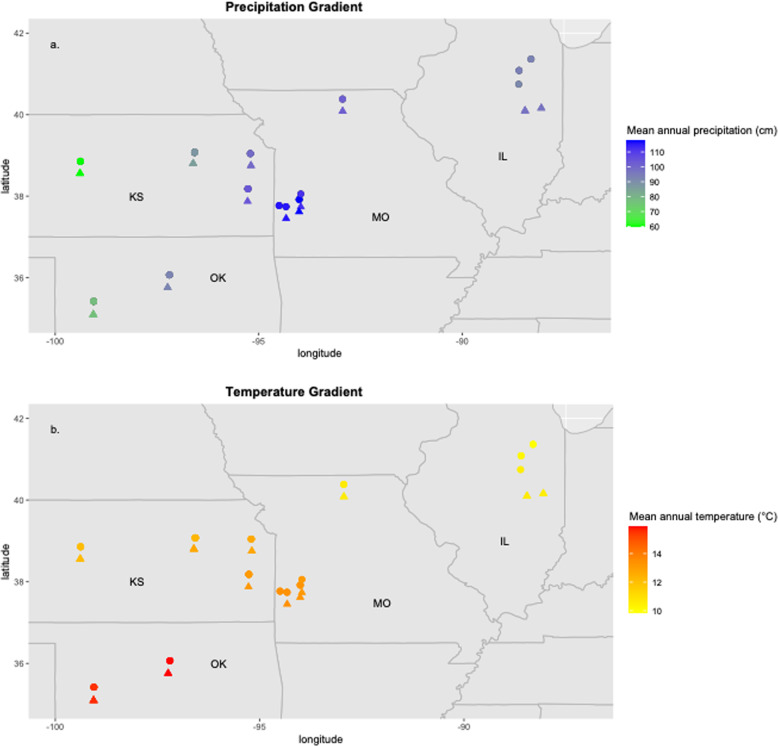
Naturally occuring precipitation and temperature gradients across study sites. Precipitation (**a**) and temperature (**b**) across our sampling sites. Remnant sites are indicated by filled circles, while disturbed sites are indicated by filled triangles. Sites are skewed vertically to avoid overlap to clarify where different sites are located. Color intensity represents rainfall (**a**) and temperature (**b**) intensity.

Samples were collected from paired remnant and disturbed grassland sites across the Midwestern United States (Fig. 1), from Illinois to Oklahoma. We paired remnants or clusters of remnants with nearby disturbed grasslands, totaling 14 remnant and 12 disturbed sites. Remnant grassland sites were defined by the absence of tilling or intensive grazing and were dominated by late successional native tallgrass prairie plant species, including *Andropogon gerardii, Schizachyrium scoparium, Sorghastrum nutans, Amorpha canescens, Echinachea pallida,* and *Silphium lacinatum*. Disturbed grassland sites had known histories of soil disturbance such as tillage (sites ranged from ~20 to 50 years since disturbance), and clear signs of anthropogenic disturbance, including overgrazing and dominance of non-native plant species, including *Festuca arundinaceae, Bromus inermis, Bromus. tectorum, Poa pratensis*, and *Bothriochloa ischaemum*. Remnant grasslands were generally more diverse than disturbed grasslands ($\overline x$ = 18.8 versus 7.5 plant species per plot, respectively). We sampled four plots arbitrarily located within each site. Four soil cores (width 2 cm, depth 15 cm) were collected arbitrarily within each of the four quadrants of each 1 m^2^ plot and composited into one sample for sequence analysis. Fine roots were collected from each sample, soil removed by hand, and frozen until DNA extraction. Soil chemical analyses including pH, Bray 1 phosphorus, and micronutrients (Melich 3) as well as Bray 2 phosphorus and C/N (Dumas method), were also conducted for most soil samples (A & L Great Lakes Labs, Fort Wayne, Indiana). Climate variables including mean annual temperature and mean annual precipitation were extracted from National Weather locations closest to each site [30]. Soil chemical analyses results and climate variables can be found in Table S1.

### Library preparation and sequencing

DNA was extracted from 35 mg of each root sample using the PowerSoil Kit (Qiagen, Hilden Germany). PCR amplification targeted the internal transcribed spacer section (ITS) of ribosome encoding genes for both fungi and oomycetes. Forward primer fITS7 [36] and reverse primer ITS4 [37] were used to amplify the ITS2 region. This region is a universal barcode for fungi [38] and is particularly suited for short Illumina MiSeq sequencing [39]. PCR amplification for oomycetes was done using recently developed oomycete-specific primers in the ITS2 region [40]: ITS3oo (Forward, AGTATGYYTGTATCAGTG) and ITS4 (Reverse, TCCTCCGCTTATTGATATGC). PCR products were visually checked on agarose gels to ensure successful amplification and cleaned using Agencourt AMPure XP magnetic beads (Beckman Coulter, Indianapolis, USA).

The fungal PCR reaction was performed using the following reactants per sample: 0.5 µl of each primer, 1 µl of extracted DNA template, 12.5 µl Phusion mastermix with HF buffer (New England Biolabs, Ipswich, MA) and 10.5 µl ddH_2_O. We used the following thermocycler program for fungal PCR: 5 min at 94 °C, 35 cycles of (30 s at 94 °C, 30 s at 57c, 30 s at 72 °C), and a final 7 min extension step at 72 °C. Fungal PCR resulted in amplicons of ~700 bases including primers and Illumina adapters. Oomycete PCR was performed using the following reactants in each sample: 0.5 µl of both primers, 1.0 µl of DNA template, 5.0 µl HOT FIREpol (Solis Biodyne, Tartu, Estonia), and 18 µl of ddH_2_O. For oomycete PCR, we used the following thermocycler program: 5 min at 95 °C, 35× (30 s at 95 °C, 30 s at 55 C°, 60 s at 72 °C), and 10 min at 72 °C. The oomycete reaction created amplicons of variable length between 400 and 700 bases.

DNA libraries for each sample and target group were created using a Nextera protocol, pooled, then sequenced using Illumina Mi-Seq (Illumina, San Diego, USA). Following the first cleanup, an indexing PCR was carried out to ligate unique 8 base-pair long sequences (molecular barcodes; Illumina, San Diego, CA, USA) to each sample. The PCR was run under similar conditions as initial PCR, except 5 µl of the primary PCR amplicon was used instead of the original DNA template, and the number of cycles was reduced to 8. Secondary PCR amplicons were purified with Agencourt AMPure XP magnetic beads and DNA concentrations were assessed by Qubit 2.0 (LifeTechnologies, Carlsbad, USA). Samples were pooled in equimolar concentration to a single library for each target group (fungi and oomycetes). Fungal and oomycete sequences were generated using an Illumina Mi-Seq (Illumina, San Diego, USA) at the KU Sequencing Core (Lawrence, KS). Raw sequencing data (fastq files) are available at Sequence Read Archive, BIOPROJECT #PRJNA532765.

### Bioinformatics

Bioinformatic analysis of sequencing data used an operational taxonomic unit (OTU) approach through the Qiime pipeline, followed by taxonomic, ecological group and phylogenetic assignment. Sequencing data were analyzed following Caporaso et al. [41] using Qiime v.1.9.1. Quality and barcode filtering resulted in 11,951,250 reads with an average phred score ≥20 and median length of 278.69 bases for fungal sequencing and 20,752,280 reads with an average phred score ≥20 and median length of 287.24 bases for oomycete sequencing. Open-reference OTU picking using sortmerna_sumaclust (pick_open_reference_otus.py) and the UNITE fungal ITS reference database v7 [42] or a custom curated oomycete reference database (available upon request) were used to cluster OTUs at 97% similarity. All OTUs with <5 reads overall were removed to eliminate potential PCR/sequencing artefacts, as recommended by Lindahl et al. [43]. All data were normalized using DESeq2 implemented in Qiime [44], using the normalize_table.py script before analysis. In total, there were 866 fungal pathogen, 3595 oomycete, and 3414 fungal saprotroph OTUs we could identify in this study. Saturation curves for each analyzed group show that more diversity is present in our system than identified here (Fig. S1). The entire bioinformatics pipeline and OTU tables are available upon request.

To identify putative fungal plant root-associated pathogens from the broader fungal OTUs, we assigned taxonomy from UNITE using RDP [45]. Then, because pathogenicity arose independently in multiple fungal lineages [46] and therefore pathogens are often closely related to nonpathogenic species, we contrasted the resulting taxonomic identities against the FUNGuild database [47]. Overall, 15.4% of fungal taxa were assignable to functional guild using FUNGuild (Table S2). We identified putative fungal pathogens within this group based on a FUNGuild assignment that contained “pathotroph” and were categorized with confidence of either highly probable or probable (17.8%, Table S2b). In this way, the fungal pathogen assignment was liberal to ensure that fungi which can be pathogens in certain environments were not excluded. Although FUNGuild and other existing databases are incomplete, our analyses that use these databases to identify taxa and putative fungal pathogens are robust to assess our hypotheses on climate and land use. One might expect pathogens from disturbed sites to be overrepresented in these databases, as the majority of plant-pathogen work has historically been agricultural, but we find little evidence for this bias in identification between remnant (11.4%) and disturbed (13.6%) sites. In addition, fungal saprotrophs were identified using FUNGuild as described above for fungal pathogens to assess whether fungal pathogen responses match those of other fungi identified through this process (Table S2). For oomycetes, we checked the identity of resulting OTUs either against a database containing all NCBI oomycote ITS2 sequence results using the Basic Local Alignment Search Tool, BLAST v. 2.6.0 [48], using default parameters, or through placing OTUs in the oomycete clade, as the oomycota are thought to have arisen from a common ancestor forming a conserved clade [49] and generally function as pathogens [27, 28].

### Statistical analysis

All statistical analyses were carried out on two plant pathogen groups: fungal pathogens and oomycetes. In addition, we analyzed fungal OTUs identified as saprotrophs (decomposers) to compare with fungal pathogen results. We ran all analyses for phylogenetically and BLAST determined oomycete OTUs, but because oomycete OTUs were not as effectively identified by BLAST, we report phylogenetic oomycete results here (BLAST results can be found in Supplementary Information for both generalized linear mixed effect model (GLM) (Table S3) and PERMANOVA analyses (Table S4)).

We tested the impact of disturbance, temperature, and precipitation (alongside other edaphic variables) on phylogenetic species richness (PSR-see below; GLM), and community composition (PERMANOVA). We then assessed differential presence (Venn diagrams) and abundance (DESeq2) of each OTU between undisturbed and disturbed grasslands. All statistical analyses were carried out in R version 3.4.1 [50].

### Estimating phylogenetic richness

PSR, [51] accounts for phylogenetic distance among taxa by using branch lengths extracted from a phylogenetic tree. We used RAxML to create our phylogenetic trees [52]. However, the evolution rate of the ITS region is relatively fast [53] and thus is not suitable to build a global tree to assess the PSR of fungal pathogens or saprotrophs. Instead, we built a family-level tree from the small ribosomal subunit using the kingdom-level fungal tree based on six genes as a backbone constraint [46]. We then manually edited the phylogenetic matrix to include the number of ITS2 identified OTUs per family, setting the distance between OTUs in the same family at 0.05, a small number relative to the distance between neighboring families. While this assumption limits the information on relationships within family, this approach represents the major advantage of PSR, which is sensitive to the distribution of OTUs across the deeper nodes of the tree. With both trees constructed, we used the pez package [54] in R to extract PSR values. The fungal outgroup used to root our phylogenetic tree was *Rhizopus oryzae* [55]. For the oomycetes, no reference tree is available, so we constructed a tree from the ITS sequences using two outgroups: *Phaeodactylum tricornutum* and *Thalassiosira pseudonana* [49].

### Analysis of PSR differences

We used GLMs to test whether disturbance (remnant or disturbed) and environmental variables explained differences in fungal pathogen and oomycete PSR across [1] all sites, then separately across [2] remnant sites and [3] disturbed sites. We ran these separate analyses for remnant and disturbed sites to further explore significant disturbance by environmental variable interactions present in the all sites model. For the “all-sites” models, we ran linear models testing mean annual precipitation, mean annual temperature, and their individual interactions with land use. Within the separate remnant and disturbed sites only data, we ran separate linear regressions testing mean annual precipitation, mean annual temperature, and the interaction between mean annual precipitation and temperature. For the “all-sites” models, we nested disturbance within site (random effect, intercept) and for models for remnant or disturbed sites included site as a random effect to properly account for nonindependence of replicate samples within site. If we assume that our sites are representative of each type of land use (which they were to the best of our ability), identification of sites within land use as random effects allows generalization across the sampled area. Mean annual precipitation and temperature were mean-centered and scaled prior to analysis. Our variable selection was informed by literature investigating environmental predictors of soil microbial diversity [20, 26, 56] and function [19, 57].

### Analysis of differences in community composition

We used a permutational multivariate analysis of variance (PERMANOVA) to test whether disturbance (remnant or disturbed) and environmental variables explained differences in fungal pathogen and oomycete community composition, respectively, across all sites. Our environmental predictor variables included mean annual precipitation, mean annual temperature, phosphorus, calcium, potassium, and soil pH (as well as each in an interaction with land use for analysis across all sites). Because some disturbance by environmental variable interactions were significant in our model for all sites (see results), we also used a PERMANOVA to assess how environmental variables impacted pathogen community composition in remnant and disturbed sites separately. Finally, we reran these PERMANOVAs to test for an interaction between temperature and precipitation as these were our two major climate change gradients and did not covary (see Fig. 1). We stratified the PERMANOVA by each combination of disturbance and site to account for random effects due to spatial proximity of paired disturbed and remnant plots within any one site. These PERMANOVA tests were performed using Morisita’s dissimilarity index, which is robust to unequal sampling [58], and the *adonis2* function in vegan Version 2.4–6 [59].

### Analysis of differential abundance and occurrence

Finally, we analyzed the data to understand differential presence (Venn diagrams) and abundance (DESeq2; [44]) of OTUs between remnant and disturbed grasslands. We constructed Venn diagrams using VennDiagram (version 1.6.19) to determine shared and unique OTUs between disturbed and remnant grasslands. We then analyzed the data using DESeq2, which allows comparison of individual OTU’s differential abundance between two groups of sites while correcting for both variation in sequence number across samples and variance in sequence number for each OTU [60]. We binned sites into low (<800 mm annual precipitation) and high (<800 mm) levels of precipitation, with Western sites representing low precipitation, and Eastern sites representing high precipitation. We then used DESeq2 to examine turnover between these Western and Eastern sites within remnant and disturbed sites separately. Because remnant grasslands had greater turnover across the East–West precipitation gradient, we then reran DESeq2 analysis within Western sites only and within Eastern sites only to determine variation between disturbed and remnant grasslands in these two specific regions.

## Results

In both groups of root-associated plant pathogens studied here—fungal pathogens and oomycetes—richness and community composition responded to environmental variables, in remnant, undisturbed grasslands, but showed a reduced sensitivity to environmental variation in disturbed grasslands.

### Phylogenetic richness

**Fig. 2:**
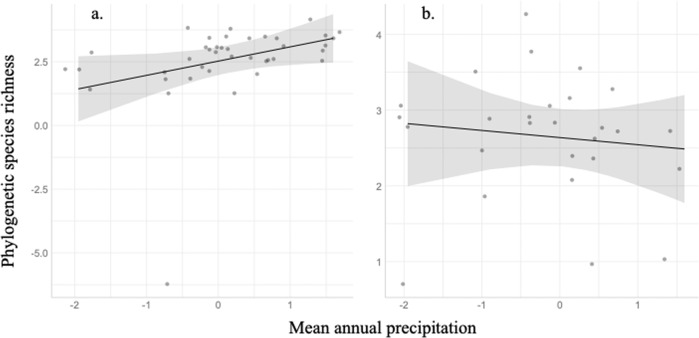
Precipitation predicts fungal pathogen phylogenetic species richness in remnant grasslands. GLM results showing mean annual precipitation prediction of phylogenetic species richness in fungal pathogens (**a**., remnant *p* = 0.06, **b**., disturbed *p* = 0.62). Points represent the raw data; the trendline is the predicted probability from the GLM.

**Fig. 3:**
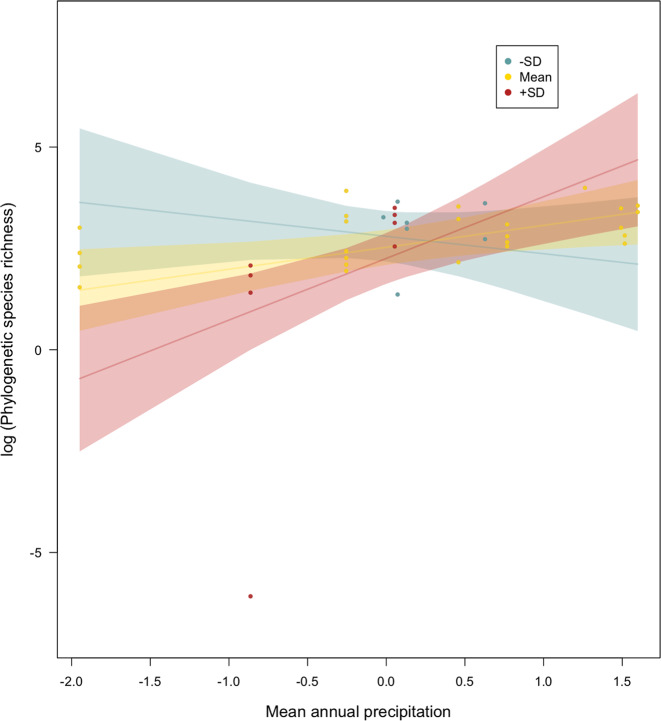
Precipitation and temperature interact to predict fungal pathogen phylogenetic species richness in remnant grasslands. Soil fungal pathogen richness depends on the interaction between precipitation and temperature in remnant grasslands (Table 1b, *p* = 0.02). Pathogen phylogenetic species richness increases with precipitation at higher temperature, but decreases with precipitation at lower temperature.

For fungal pathogens, PSR was predicted by environmental variables, particularly precipitation, in remnant (Table 1b; *F*_1,13.55_ = 4.26, *p* = 0.06), but not in disturbed grasslands (Fig. 2, Table 1b). In remnant grasslands only, precipitation and temperature interacted to determine PSR, with precipitation associated with greater fungal pathogen PSR when temperature was higher, but not when temperature was lower (Fig. 3; Table 1b; *F*_1,36_ = 6.22, *p* = 0.02). Oomycetes showed similar responses, with oomycete PSR in remnant grasslands increased with precipitation (Table 1; *F*_1,17.75_ = 4.62, *p* = 0.05), but in disturbed grasslands oomycete PSR was unrelated to environmental variables.

**Table 1:** PSR richness GLM results for oomycetes (a), fungal pathogens (b), and fungal saprotrophs (c).

Subset of samples	Predictor variables	Estimate	*p* value
(a) Oomycetes			
All samples	Disturbance	−0.022	0.389
	Disturbance × Mean annual precipitation	0.004	0.863
	Disturbance × Mean annual temperature	−0.036	0.183
Remnant samples only	Mean annual precipitation	0.033	**0.046**
	Mean annual temperature	−0.012	0.494
	Precipitation × Temperature	−0.03	0.233
Disturbed samples only	Mean annual precipitation	0.029	0.123
	Mean annual temperature	0.024	0.257
	Precipitation × Temperature	−0.002	0.951
(b) Fungal pathogens			
All samples	Disturbance	−0.005	0.990
	Disturbance × Mean annual precipitation	0.645	0.080
	Disturbance × Mean annual temperature	−0.369	0.326
Remnant samples only	Mean annual precipitation	0.559	**0.059**
	Mean annual temperature	−0.386	0.199
	Precipitation × Temperature	0.990	**0.017**
Disturbed samples only	Mean annual precipitation	−0.094	0.620
	Mean annual temperature	−0.018	0.921
	Precipitation × Temperature	−0.282	0.412
(c) Fungal saprotrophs			
All samples	Disturbance	−0.353	0.314
	Disturbance × Mean annual precipitation	0.366	0.291
	Disturbance × Mean annual temperature	−0.090	0.748
Remnant samples only	Mean annual precipitation	0.415	0.126
	Mean annual temperature	−0.524	**0.035**
	Precipitation × Temperature	1.033	**0.001**
Disturbed samples only	Mean annual precipitation	0.050	0.817
	Mean annual temperature	−0.444	**0.001**
	Precipitation × Temperature	−0.041	0.840

These tests are univariate tests including either the interaction of the predictor variable and disturbance (if across all sites), or only the predictor variable in determining PSR richness. Tests of all samples include a random effect of disturbance nested within site; tests of remnant or disturbed samples include a random effect of plot. The model distribution is poisson. All significant predictors are in bold.

### Differences in community composition

Anthropogenic disturbance of grasslands, as well as precipitation and temperature, influenced fungal pathogen and oomycete composition (Table 2; disturbance and precipitation, *p* < 0.001; temperature *p* < 0.01). As with richness, environmental factors predicted soil pathogen community composition in remnant grasslands, but this sensitivity was reduced in disturbed sites (Table 2). We found a significant temperature by precipitation interaction in fungal pathogens in both remnant and disturbed sites (Table 2b, remnant: *p* = 0.04, *R*^2^ = 0.049; disturbed: *p* = 0.03, *R*^2^ = 0.099). In remnant sites, the significant environmental factors explained a total of 39 percent of variation, while in disturbed sites they explained 10 percent of variation; although the precipitation by temperature interaction is significant in both disturbance groups, edaphic responses were absent, leading to a much lower impact of environmental variables on community composition in disturbed sites. Mean annual temperature and calcium were significant predictors of remnant community composition in both fungal pathogens and oomycetes (Table 2; fungal pathogens: temperature: *p* < 0.01, *R*^2^ = 0.074; calcium: *p* = 0.01, *R*^2^ = 0.07; oomycetes: temperature: *p* = 0.03, *R*^2^ = 0.056, calcium: *p* = 0.04, *R*^2^ = 0.050). Fungal pathogen remnant community composition was also significantly predicted by phosphorous, soil pH and potassium (Table 2; phosphorus: *p* < 0.01, *R*^2^ = 0.073; soil pH: *p* < 0.01, *R*^2^ = 0.070; potassium: *p* = 0.03, *R*^2^ = 0.056).

**Table 2:** PERMANOVA results for phylogenetic oomycetes (a) and fungal pathogens (b).

Subset of samples	Predictor variables	*R*^2^ value	*p* value
(a) Oomycetes			
All samples	Disturbance	0.061	**0.000**
	Mean annual precipitation	0.092	**0.000**
	Mean annual temperature	0.044	**0.001**
	Bray 2 phosphorus	0.054	**0.000**
	Potassium	0.035	**0.007**
	Calcium	0.029	**0.023**
	Soil pH	0.012	0.621
	Disturbance × Mean annual precipitation	0.038	**0.003**
	Disturbance × Mean annual temperature	0.019	0.188
	Disturbance × Bray 2 phosphorus	0.024	0.052
	Disturbance × Potassium	0.022	0.093
	Disturbance × Calcium	0.025	**0.042**
	Disturbance × Soil pH	0.020	0.153
	Sequence number	0.053	**0.000**
Remnant samples only	Mean annual precipitation	0.030	0.314
	Mean annual temperature	0.056	**0.027**
	Bray 2 phosphorus	0.048	0.050
	Potassium	0.037	0.137
	Calcium	0.050	**0.043**
	Soil pH	0.043	0.084
	Sequence number	0.052	**0.030**
	Mean annual precipitation × Temperature	0.023	0.879
Disturbed samples only	Mean annual precipitation	0.061	0.109
	Mean annual temperature	0.064	0.100
	Bray 2 phosphorus	0.062	0.109
	Potassium	0.060	0.122
	Calcium	0.066	0.090
	Soil pH	0.054	0.175
	Sequence number	0.044	0.309
	Mean annual precipitation × Temperature	0.048	0.203
(b) Fungal pathogens			
All samples	Disturbance	0.065	**0.000**
	Mean annual precipitation	0.096	**0.000**
	Mean annual temperature	0.055	**0.001**
	Bray 2 phosphorus	0.050	**0.002**
	Potassium	0.056	**0.001**
	Calcium	0.011	0.671
	Soil pH	0.018	0.274
	Disturbance × Mean annual precipitation	0.028	0.057
	Disturbance × Mean annual temperature	0.016	0.344
	Disturbance × Bray 2 phosphorus	0.014	0.473
	Disturbance × Potassium	0.018	0.300
	Disturbance × Calcium	0.016	0.403
	Disturbance × Soil pH	0.035	**0.017**
	Sequence number	0.027	0.057
Remnant samples only	Mean annual precipitation	0.050	0.053
	Mean annual temperature	0.074	**0.005**
	Bray 2 phosphorus	0.073	**0.006**
	Potassium	0.056	**0.029**
	Calcium	0.067	**0.011**
	Soil pH	0.070	**0.007**
	Sequence number	0.023	0.463
	Mean annual precipitation × Temperature	0.049	**0.042**
Disturbed samples only	Mean annual precipitation	0.044	0.455
	Mean annual temperature	0.050	0.400
	Bray 2 phosphorus	0.049	0.406
	Potassium	0.046	0.432
	Calcium	0.052	0.379
	Soil pH	0.045	0.444
	Sequence number	0.056	0.272
	Mean annual precipitation × Temperature	0.099	**0.028**
(c) Fungal saprotrophs			
All samples	Disturbance	0.099	**0.000**
	Mean annual precipitation	0.169	**0.000**
	Mean annual temperature	0.053	**0.000**
	Bray 2 phosphorus	0.081	**0.000**
	Potassium	0.033	**0.006**
	Calcium	0.035	**0.003**
	Soil pH	0.022	0.066
	Disturbance × Mean annual precipitation	0.016	0.195
	Disturbance × Mean annual temperature	0.014	0.270
	Disturbance × Bray 2 phosphorus	0.021	0.067
	Disturbance × Potassium	0.010	0.536
	Disturbance × Calcium	0.015	0.265
	Disturbance × Soil pH	0.020	0.087
	Sequence number	0.012	0.439
Remnant samples only	Mean annual precipitation	0.023	0.329
	Mean annual temperature	0.052	**0.013**
	Bray 2 phosphorus	0.050	**0.015**
	Potassium	0.033	0.097
	Calcium	0.052	**0.012**
	Soil pH	0.047	**0.021**
	Sequence number	0.026	0.219
	Mean annual precipitation × Temperature	0.025	0.238
Disturbed samples only	Mean annual precipitation	0.039	0.404
	Mean annual temperature	0.037	0.479
	Bray 2 phosphorus	0.037	0.470
	Potassium	0.040	0.402
	Calcium	0.031	0.597
	Soil pH	0.048	0.210
	Sequence number	0.036	0.415
	Mean annual precipitation × Temperature	0.039	0.340

Separate tests were run within each oomycetes (a), fungal pathogens (b) and fungal saprotrophs (c) for all, remnant and disturbed sites; a second set of analyses was run for remnant and disturbed sites to test for the interaction between temperature and precipitation. All significant predictors are in bold.

### Differential abundance and occurrence

There were a greater number of unique pathogen OTUs present in remnant versus disturbed grasslands as found in the Venn diagrams (Fig. S2); this was especially striking for oomycetes (BLAST) with over double the unique OTUs (1555) compared to disturbed grasslands (628). Comparison of the relative abundance of OTUs via DESeq2 confirms greater turnover in remnant than in disturbed sites across the precipitation gradient (west versus east; Fig. S3). Because of the divergent composition across remnant grasslands, we compared differential abundance of OTUs in remnant versus disturbed grasslands in eastern and western sites separately. Remnant sites tended to have fewer differentially abundant OTUs (between eastern and western sites) than disturbed sites when analyzing fungal pathogens and oomycetes (Fig. S4). Although there are fewer OTUs in disturbed grasslands, a greater proportion of these OTUs are differentially abundant in disturbed grasslands compared to remnant grasslands, suggesting that they could be disturbance specialists.

### Fungal saprotrophs

Fungal pathogens and saprotrophs differed in diversity responses to climate and land use, but had similar community composition responses. While climate factors only predicted pathogen PSR in remnant sites, climate predicted saprotroph PSR in both remnant and disturbed sites (Table 1c; remnant: *F*_1,11.7_ = 5.67, *p* = 0.04; disturbed: *F*_1,25_ = 13.46, *p* < 0.01). Similar to pathogens, the interaction of precipitation and temperature predicted saprotroph PSR (Table 1c; *F*_1,36_ = 13.73, *p* < 0.01; Fig S5) in remnant grasslands. Precipitation was positively correlated to PSR when temperature was high, but not when temperature was low. Saprotroph community composition responses mirrored those found for fungal pathogens, with several significant climate predictors for remnant, but none for disturbed (Table 2c). Therefore, disturbance had distinct effects on climate relationships for pathogen richness as compared to saprotrophs, but showed similar results in terms of community composition.

## Discussion

In a comprehensive test of root-associated pathogen sensitivity to environmental factors, we find that the community structure of fungal pathogens and oomycetes changes with anthropogenic disturbance. Moreover, we find that root-associated fungal and oomycete pathogen communities are sensitive to climate gradients, particularly precipitation and temperature, in undisturbed grasslands, but that disturbance disrupts the responses of these root-associated plant pathogens to environmental factors. As with other recent work, edaphic factors play an important role in structuring these grassland fungal communities [15–19, 56]. Together, these results identify interactive effects of climate and disturbance on plant pathogen communities, with implications for understanding potential patterns of the impact of pathogens on plant community composition and diversity.

### Climatic determinants of root-associated plant pathogen communities in remnant grasslands

In the absence of disturbance, the structure of root-associated plant pathogen communities in remnant grasslands changes with climatic factors, including both precipitation and temperature. In contrast, most existing literature on fungal communities find either that climatic variables are not important to community structure [16, 17] or these variables are not explored [15, 56]. Our work adds to the growing evidence that soil fungi in general respond to climatic factors in addition to edaphic properties [18, 20, 25]. For example, Zhou et al. [20] investigated six forests across northern and central America, representing a 30 °C temperature gradient and found that fungal diversity was better predicted by variation in temperature than edaphic properties. Likewise, Rincón et al. [18] showed that fungal community composition responded to temperature and precipitation across a set of scots pine forests in France and Spain. Recent work by Spear [25] showed that diversity of putative fungal pathogens from leaf stem and root tissue isolated on media responds positively to precipitation across a natural rainfall gradient in Panama. Our study is the first to show similar patterns for root-associated pathogens in undisturbed grasslands.

Oomycetes also respond to precipitation in remnant grasslands, perhaps due to their life history. For example, oomycete zoospore release and subsequent flagellar movement to find a host explicitly depend on wet conditions [28]. Precipitation has previously been shown to be an important driver of oomycete community composition, although most studies showing this were conducted in agricultural settings [27]. In agreement with a recent global analysis of oomycete environmental drivers showing the positive relationship between precipitation and oomycete abundance [23], oomycete richness in our study responded positively to precipitation in remnant, undisturbed grasslands.

Temperature modifies the response of fungal pathogen diversity to precipitation (i.e., temperature and precipitation interact, Fig. 3). This has important implications for predicting the impact of these two major climate variables on remaining grassland systems. In sites with especially high average temperatures, increasing precipitation corresponds to an increase in OTU richness, but this effect is absent across sites with colder average temperatures. Zhang et al. [33] also found a similar temperature by precipitation interaction using PLFAs to assess soil fungi; precipitation and temperature interacted to promote stimulation of functional groups, while under drought, this relationship disappeared. In addition, Talley et al. [24] found that vapor pressure deficit, a metric combing temperature and relative humidity, explained fungal richness better than temperature alone. In contrast, Ochoa-Hueso et al. [61] found soil fungal diversity decreases with precipitation, although these results may be a product of different temperature regimes. While this interaction has been shown for bacteria [34] and fungi in general [33], our results are, to our knowledge, the first to show it for root-associated fungal pathogens. Given predicted shifts in both temperature and precipitation due to climate change, experimental assessment of the interactions between these factors is sorely needed.

### Anthropogenic disturbance shifts root-associated plant pathogen composition and alters dependence on climate

Anthropogenic disturbance impacted pathogen community composition. The community composition of both oomycetes and fungal pathogens differed in disturbed compared to remnant grasslands. Our results are consistent with other studies showing strong effects of land use in non-pathogenic microbes [30, 62]. We note that these differences in pathogen composition persisted at some sites decades after disturbance ended. One might ask why this difference has persisted so long. It is quite possible that there were few opportunities for dispersal of native pathogens to disturbed grasslands, as the vast majority of remnant grassland has been destroyed by tillage over the last hundred years of agriculture. With remnant grasslands occurring in less than four percent of original extent [63], there are very few remaining sources of native microbes, including pathogens, and these sources could be many miles from the disturbed grasslands we sampled. Alternatively, the successful colonization of disturbed grasslands by native plant pathogens could be limited by other persistent legacies of anthropogenic disturbance. As anthropogenic disturbance includes tillage and fertilization effects, its impact on the microbial composition could be mediated by changes in soil structure or fertility. Edaphic mediation of disturbance effects has been observed on nonpathogenic microbial groups [16, 56]. However, our data show a consistent differentiation of root-associated pathogen community structure between remnant and disturbed sites independent of measured edaphic properties. It is also possible, and perhaps likely, that the persistent change in plant composition following disturbance could contribute to these shifts in pathogen community composition. The disturbed grasslands sampled here had a markedly different plant composition, including dominance by non-native plant species, compared to remnant grasslands. Because plant community composition overlapped so little between remnant and disturbed sites (with many disturbed sites having no overlap in plant species composition with remnant sites), linking pathogen shifts to individual plant species differences was not possible. However, given host-specificity of plant pathogens [2, 64], it is likely that the loss of the native prairie plant species in disturbed grasslands would limit establishment success of pathogens from remnant grasslands.

We also find that plant pathogens in anthropogenically disturbed grasslands are less responsive to variation in climate than in remnant grasslands, compared to other functional groups. Our results indicate a disturbance-induced reduction in climate sensitivity of root-associated pathogens. Increased homogenization of both soil properties and plant communities in disturbed grasslands likely leads to a prevalence of shared, disturbance-adapted pathogens across the sites. Fungal saprotroph community composition was also linked to climate in remnant, but not disturbed grasslands. Unlike fungal pathogens and oomycetes, however, climate factors predicted PSR of fungal saprotrophs in both remnant and disturbed systems. In previous work, arbuscular mycorrhizal fungi (AMF) communities in remnant sites also differed in response to precipitation, similar to patterns we find in pathogens, but AMF richness was not affected by climate [30]. Despite some similarities across functional groups, our data support distinct OTU richness responses of pathogenic fungi, saprotrophic fungi, and AMF to disturbance and climate. Given their different functional roles, these data support expectations that different groups within the microbial community can react differently to climatic and land use drivers.

Our results support the hypothesis that pathogen communities in undisturbed native grasslands are more responsive to precipitation and temperature than disturbed grasslands, yet we urge careful interpretation of these results. Taxonomic and functional group assignment rely on well-informed reference databases, which may be lacking particularly in remnant, undisturbed systems. The common practice of removing all OTUs that do not match a database (e.g., BLAST) may skew results toward cultured, heavily studied, or economically important organisms, such as those found in agricultural settings. For example, 509 oomycete taxa were excluded from our initial OTU table based on matching BLAST sequences because of the limited reference database available. Phylogenetic taxa delineation (used here) rather than a BLAST approach is more appropriate for the poorly described pathogens of native communities and generated a larger pool of resident oomycetes. Functional variation may also impact our conclusions. For example, the assumption that all oomycota are pathogens is widely supported. Certain oomycetes, however, have been shown to be saprophytic instead of pathogenic [28] and may have a spectrum of pathogen and saprotrophic potential [65]. Likewise, our liberal inclusion of fungi designated by FUNGuild as pathogens likely masks a spectrum of functional variation. Inclusion of only high confidence designations, similar designations among site types, and comparison to FUNGuild-designated saprotrophs supports that these results are not products of database bias alone. Ideally, a more complete, experimental assessment of pathogenicity among oomycetes and fungal pathogens might allow more accurate ecological inferences about these groups across grasslands.

### Study implications

Our findings have implications for restorations of disturbed grasslands as well as remnant grasslands that have undergone the effects of climate change. To the extent that pathogens contribute to the maintenance of plant diversity [2], degradation of pathogen diversity and composition could contribute to the reduced plant diversity often observed following anthropogenic disturbance [63, 66, 67]. Successful restoration of native plant diversity in grasslands may depend on reintroduction of these lost pathogens. In undisturbed systems, greater precipitation increases pathogen diversity, both for oomycetes and fungal pathogens, potentially contributing to increased native plant diversity. Within a changing climate, however, focusing solely on precipitation may not effectively predict these microbial communities, since precipitation effects here depended on temperature. While we cannot separate the direct effects of climate on pathogen composition from those effects mediated through plant responses, our results suggest that incorporation of environmental sensitivities of pathogens may be important to long-term predictions of plant community response to climate. Further work is necessary to understand the causes and consequences of the precipitation and temperature interaction in pathogen groups to enact effective management strategies.

## Conclusion

In conclusion, our study shows that different groups of root-associated plant pathogenic microbes are sensitive to land use disturbance and environmental gradients. Environmental gradients are important in driving pathogen community responses in undisturbed remnant, but less so in disturbed, grasslands. By clarifying root-associated plant pathogen response to temperature and precipitation gradients, we highlight the indirect consequences that climate shifts may have on plants through their microbiome. The root-associated plant pathogens studied here represent an often-overlooked mediator of plant community composition and diversity. Therefore, a clear understanding of how the plant microbiome responds to climate change will help us secure the future of remaining native plant communities and improve restoration of degraded ones.

## Supplementary information

Supplementary Information
