# Supplementary material for: Root pathogen diversity and composition varies with climate in undisturbed grasslands, but less so in anthropogenically disturbed grasslands
Source: ISME J. 2020 Sep 21;15(1):304–17. doi: 10.1038/s41396-020-00783-z (PMC7852655; doi:10.1038/s41396-020-00783-z)

**Supplementary Information**

Title: Root pathogen diversity and composition varies with climate in undisturbed grasslands, but less so in anthropogenically-disturbed grasslands

Authors: Camille S. Delavaux^1,2*^, Josh L. Schemanski^1,2^, Geoffrey L. House^3^, Alice G. Tipton^4^, Benjamin Sikes^1,2^, James D. Bever^1,2^

Affiliations:

*^1^Department of Ecology and Evolutionary Biology, The University*

*of Kansas, 2041 Haworth Hall, 1200 Sunnyside Avenue, Lawrence, Kansas 66045, USA*

*^2^Kansas Biological Survey, The University*

*of Kansas, 106 Higuchi Hall, 2101 Constant Ave, Lawrence, Kansas 66047, USA*

*^3^ National Ecological Observatory Network, Boulder, CO 80301, USA*

*^4^Department of Science, Technology, and Mathematics, Lincoln University, 821 Taylor Drive, 205 Daniel Hall, Jefferson City, Missouri 65101, USA*

Corresponding author: Camille S. Delavaux

e-mail address: [camille.delavaux@ku.edu](mailto:camille.delavaux@ku.edu)

alternative e-mail address: camzdel@gmail.com

Telephone: 908-892-9717

**Table S1.** Soil chemical analyses results and climate variables for this study.

| Sample | Site | State | Disturbance | Lat | Long | MeanTemp | Annual Precip | Bray2P | K | soil  pH |
| --- | --- | --- | --- | --- | --- | --- | --- | --- | --- | --- |
| 2 | Klemme | OK | Disturbed | 35.40 | -99.06 | 15.60 | 77.18 | NA | NA | NA |
| 3 | Klemme | OK | Disturbed | 35.40 | -99.06 | 15.60 | 77.18 | NA | NA | NA |
| 4 | Klemme | OK | Disturbed | 35.40 | -99.06 | 15.60 | 77.18 | NA | NA | NA |
| 5 | Hays | KS | Disturbed | 38.86 | -99.39 | 12.08 | 60.00 | 20 | 317 | 7.5 |
| 6 | Hays | KS | Disturbed | 38.86 | -99.39 | 12.08 | 60.00 | 20 | 317 | 7.5 |
| 7 | Hays | KS | Disturbed | 38.86 | -99.39 | 12.08 | 60.00 | 20 | 317 | 7.5 |
| 8 | Hays | KS | Disturbed | 38.86 | -99.39 | 12.08 | 60.00 | 20 | 317 | 7.5 |
| 9 | Welda | KS | Disturbed | 38.18 | -95.27 | 13.16 | 104.11 | NA | NA | NA |
| 10 | Welda | KS | Disturbed | 38.18 | -95.27 | 13.16 | 104.11 | NA | NA | NA |
| 11 | Konza | KS | Disturbed | 39.10 | -96.61 | 12.70 | 85.81 | 19 | 296 | 6.2 |
| 12 | Konza | KS | Disturbed | 39.10 | -96.61 | 12.70 | 85.81 | 19 | 296 | 6.2 |
| 13 | Klemme | OK | Disturbed | 35.40 | -99.06 | 15.60 | 77.18 | NA | NA | NA |
| 14 | Konza | KS | Disturbed | 39.10 | -96.61 | 12.70 | 85.81 | 19 | 296 | 6.2 |
| 16 | Osage | MO | Disturbed | 37.75 | -94.33 | 13.39 | 115.91 | 10 | 61 | 7.1 |
| 17 | Osage | MO | Disturbed | 37.75 | -94.33 | 13.39 | 115.91 | 10 | 61 | 7.1 |
| 18 | Taberville | MO | Disturbed | 38.04 | -93.97 | 13.14 | 112.46 | 10 | 61 | 7.1 |
| 19 | WahKonTah | MO | Disturbed | 37.92 | -94.01 | 13.28 | 117.53 | 10 | 61 | 7.1 |
| 20 | Morris | MO | Disturbed | 40.38 | -92.94 | 10.58 | 101.80 | 13 | 132 | 5.8 |
| 21 | Morris | MO | Disturbed | 40.38 | -92.94 | 10.58 | 101.80 | 13 | 132 | 5.8 |
| 22 | Rockefeller | KS | Disturbed | 39.05 | -95.19 | 12.73 | 99.16 | NA | NA | NA |
| 23 | Rockefeller | KS | Disturbed | 39.05 | -95.19 | 12.73 | 99.16 | NA | NA | NA |
| 24 | Klemme | OK | Remnant | 35.42 | -99.06 | 15.54 | 77.66 | 52 | 148 | 7.7 |
| 25 | Klemme | OK | Remnant | 35.42 | -99.06 | 15.54 | 77.66 | 52 | 148 | 7.7 |
| 26 | Klemme | OK | Remnant | 35.42 | -99.06 | 15.54 | 77.66 | 52 | 148 | 7.7 |
| 27 | Klemme | OK | Remnant | 35.42 | -99.06 | 15.54 | 77.66 | 52 | 148 | 7.7 |
| 28 | Klemme | OK | Remnant | 35.42 | -99.06 | 15.54 | 77.66 | 52 | 148 | 7.7 |
| 29 | Klemme | OK | Remnant | 35.42 | -99.06 | 15.54 | 77.66 | 52 | 148 | 7.7 |
| 30 | Klemme | OK | Remnant | 35.42 | -99.06 | 15.54 | 77.66 | 52 | 148 | 7.7 |
| 31 | Klemme | OK | Remnant | 35.42 | -99.06 | 15.54 | 77.66 | 52 | 148 | 7.7 |
| 32 | Hays | KS | Remnant | 38.86 | -99.38 | 12.10 | 60.07 | 9 | 248 | 7.7 |
| 33 | Hays | KS | Remnant | 38.86 | -99.38 | 12.10 | 60.07 | 9 | 248 | 7.7 |
| 34 | Hays | KS | Remnant | 38.86 | -99.38 | 12.10 | 60.07 | 9 | 248 | 7.7 |
| 35 | Hays | KS | Remnant | 38.86 | -99.38 | 12.10 | 60.07 | 9 | 248 | 7.7 |
| 36 | Hays | KS | Remnant | 38.86 | -99.38 | 12.10 | 60.07 | 9 | 248 | 7.7 |
| 37 | Konza | KS | Remnant | 39.07 | -96.57 | 12.28 | 87.51 | 9 | 299 | 6.3 |
| 38 | Konza | KS | Remnant | 39.07 | -96.57 | 12.28 | 87.51 | 9 | 299 | 6.3 |
| 39 | Konza | KS | Remnant | 39.07 | -96.57 | 12.28 | 87.51 | 9 | 299 | 6.3 |
| 40 | Konza | KS | Remnant | 39.07 | -96.57 | 12.28 | 87.51 | 9 | 299 | 6.3 |
| 41 | Rockefeller | KS | Remnant | 39.05 | -95.21 | 12.67 | 99.07 | 8 | 141 | 6.2 |
| 42 | Rockefeller | KS | Remnant | 39.05 | -95.21 | 12.67 | 99.07 | 8 | 141 | 6.2 |
| 43 | Rockefeller | KS | Remnant | 39.05 | -95.21 | 12.67 | 99.07 | 8 | 141 | 6.2 |
| 44 | Welda | KS | Remnant | 38.18 | -95.27 | 13.14 | 104.08 | 2 | 76 | 6.1 |
| 45 | Welda | KS | Remnant | 38.18 | -95.27 | 13.14 | 104.08 | 2 | 76 | 6.1 |
| 46 | Rockefeller | KS | Remnant | 39.05 | -95.21 | 12.67 | 99.07 | 8 | 141 | 6.2 |
| 47 | Welda | KS | Remnant | 38.18 | -95.27 | 13.14 | 104.08 | 2 | 76 | 6.1 |
| 48 | Weston | IL | Remnant | 40.75 | -88.61 | 10.49 | 91.34 | NA | NA | NA |
| 49 | Weston | IL | Remnant | 40.75 | -88.61 | 10.49 | 91.34 | NA | NA | NA |
| 50 | Sunbury | IL | Remnant | 41.08 | -88.60 | 10.21 | 93.75 | NA | NA | NA |
| 51 | Sunbury | IL | Remnant | 41.08 | -88.60 | 10.21 | 93.75 | NA | NA | NA |
| 52 | Goose_Lake | IL | Remnant | 41.36 | -88.31 | 9.92 | 92.80 | NA | NA | NA |
| 53 | Goose_Lake | IL | Remnant | 41.36 | -88.31 | 9.92 | 92.80 | NA | NA | NA |
| 54 | Konza | KS | Remnant | 39.07 | -96.57 | 12.28 | 87.51 | 9 | 299 | 6.3 |
| 55 | Konza | KS | Remnant | 39.07 | -96.57 | 12.28 | 87.51 | 9 | 299 | 6.3 |
| 56 | Konza | KS | Remnant | 39.07 | -96.57 | 12.28 | 87.51 | 9 | 299 | 6.3 |
| 57 | Konza | KS | Remnant | 39.07 | -96.57 | 12.28 | 87.51 | 9 | 299 | 6.3 |
| 58 | Welda | KS | Remnant | 38.18 | -95.27 | 13.14 | 104.08 | 2 | 76 | 6.1 |
| 59 | Big_Osage | MO | Remnant | 37.74 | -94.33 | 13.38 | 115.81 | 2 | 28 | 5.7 |
| 60 | Big_Osage | MO | Remnant | 37.74 | -94.33 | 13.38 | 115.81 | 2 | 28 | 5.7 |
| 61 | Little_Osage | MO | Remnant | 37.77 | -94.34 | 13.39 | 116.19 | 2 | 28 | 5.7 |
| 62 | Little_Osage | MO | Remnant | 37.77 | -94.34 | 13.39 | 116.19 | 2 | 28 | 5.7 |
| 63 | WahKonTah | MO | Remnant | 37.92 | -94.01 | 13.28 | 117.53 | 8 | 78 | 5.9 |
| 64 | WahKonTah | MO | Remnant | 37.92 | -94.01 | 13.28 | 117.53 | 8 | 78 | 5.9 |
| 65 | Taberville | MO | Remnant | 38.06 | -93.97 | 13.12 | 112.09 | 8 | 78 | 5.9 |
| 66 | Taberville | MO | Remnant | 38.06 | -93.97 | 13.12 | 112.09 | 8 | 78 | 5.9 |
| 67 | Morris | MO | Remnant | 40.38 | -92.94 | 10.58 | 101.80 | 3 | 74 | 6.3 |
| 68 | Morris | MO | Remnant | 40.38 | -92.94 | 10.58 | 101.80 | 3 | 74 | 6.3 |
| 69 | IL_Rte54 | IL | Disturbed | 40.40 | -88.46 | 10.60 | 97.10 | 19 | 223 | 6.1 |
| 70 | IL_Rte54 | IL | Disturbed | 40.40 | -88.46 | 10.60 | 97.10 | 19 | 223 | 6.1 |
| 71 | IL_Rte54 | IL | Disturbed | 40.40 | -88.46 | 10.60 | 97.10 | 19 | 223 | 6.1 |
| 72 | Paxton_East | IL | Disturbed | 40.46 | -88.06 | 10.48 | 96.87 | 25 | 230 | 7.3 |
| 73 | Paxton_East | IL | Disturbed | 40.46 | -88.06 | 10.48 | 96.87 | 25 | 230 | 7.3 |
| 74 | Paxton_East | IL | Disturbed | 40.46 | -88.06 | 10.48 | 96.87 | 25 | 230 | 7.3 |
| 75 | Stillwater | OK | Remnant | 36.07 | -97.19 | 15.88 | 92.51 | NA | NA | NA |
| 76 | Stillwater | OK | Remnant | 36.07 | -97.19 | 15.88 | 92.51 | NA | NA | NA |
| 77 | Stillwater | OK | Remnant | 36.07 | -97.19 | 15.88 | 92.51 | NA | NA | NA |
| 78 | Stillwater | OK | Remnant | 36.07 | -97.19 | 15.88 | 92.51 | NA | NA | NA |
| 79 | Stillwater | OK | Disturbed | 36.06 | -97.24 | 15.90 | 92.20 | NA | NA | NA |
| 80 | Stillwater | OK | Disturbed | 36.06 | -97.24 | 15.90 | 92.20 | NA | NA | NA |

**Table S2.** The proportion of fungi that were identified with FUNGuild from all fungal OTUs, the proportion of fungi identified in remnant versus disturbed sites with FUNGuild, and the proportion of FUNGuild fungi identified as either pathotroph or saprotroph.

| **TABLE S2** | |
| --- | --- |
| Proportion identified | |
| Proportion Identified FUNGuild | 0.154 |
| Proportion FUNGuild Identified in Remnant Samples | 0.114 |
| Proportion FUNGuild Identified in Disturbed Samples | 0.136 |
| Proportion FUNGuild Fungi Identified as Pathotroph | 0.178 |
| Proportion FUNGuild Fungi Identified as Saprotroph | 0.704 |

**Table S3.** PSR richness GLM results for BLAST oomycetes. These tests are univariate tests including either the interaction of the predictor variable and disturbance (if across all sites), or only the predictor variable in determining PSR richness. The all sample test includes a random effect of disturbance nested within site; tests of remnant or disturbed samples include a random effect of plot. The model distribution is poisson. All significant predictors are in bold.

| **TABLE S3** | | | |
| --- | --- | --- | --- |
| Subset of samples | Predictor variables | Estimate | *p* value |
| All samples | Disturbance | -0.048 | 0.647 |
|  | Disturbance × Mean Annual Precipitation | 0.094 | 0.321 |
|  | Disturbance × Mean Annual Temperature | -0.154 | 0.165 |
| Remnant samples only | Mean Annual Precipitation | 0.163 | **0.016** |
|  | Mean Annual Temperature | -0.058 | 0.421 |
|  | Precipitation × Temperature | -0.100 | 0.357 |
| Disturbed samples only | Mean Annual Precipitation | 0.072 | 0.345 |
|  | Mean Annual Temperature | 0.096 | 0.236 |
|  | Precipitation × Temperature | -0.018 | 0.893 |

**Table S4**. PERMANOVA results for BLAST oomycetes. *P < 0.05, **P < 0.01, ***P < 0.001. Separate tests were run for all, and again within remnant and disturbed sites; a second set was run for remnant and disturbed sites to test for the interaction between temperature and precipitation. We stratified the PERMANOVA by each combination of disturbance and site to account for random effects due to spatial proximity of paired disturbed and remnant plots within any one site.

| **TABLE S4** | | | |
| --- | --- | --- | --- |
| Subset of samples | Predictor variables | R^2^ value | *p* value |
| All samples | Disturbance | 0.06182 | *** |
|  | Mean Annual Precipitation | 0.09192 | *** |
|  | Mean Annual Temperature | 0.04450 | ** |
|  | Bray 2 Phosphorus | 0.05432 | *** |
|  | Potassium | 0.03397 | ** |
|  | Calcium | 0.02885 | * |
|  | Soil pH | 0.01168 |  |
|  | Disturbance × Mean Annual Precipitation | 0.03820 | ** |
|  | Disturbance × Mean Annual Temperature | 0.01787 |  |
|  | Disturbance × Bray 2 Phosphorus | 0.02282 |  |
|  | Disturbance × Potassium | 0.02194 |  |
|  | Disturbance × Calcium | 0.02223 |  |
|  | Disturbance × Soil pH | 0.01982 |  |
|  | Sequence number | 0.06689 | *** |
| Remnant samples only | Mean Annual Precipitation | 0.03030 |  |
|  | Mean Annual Temperature | 0.05460 | * |
|  | Bray 2 Phosphorus | 0.04809 |  |
|  | Potassium | 0.03817 |  |
|  | Calcium | 0.04965 | * |
|  | Soil pH | 0.04305 |  |
|  | Sequence number | 0.06206 | * |
|  | Mean Annual Precipitation × Temperature | 0.02373 |  |
| Disturbed samples only | Mean Annual Precipitation | 0.04849 |  |
|  | Mean Annual Temperature | 0.04656 |  |
|  | Bray 2 Phosphorus | 0.04582 |  |
|  | Potassium | 0.04651 |  |
|  | Calcium | 0.04713 |  |
|  | Soil pH | 0.04587 |  |
|  | Sequence number | 0.03609 |  |
|  | Mean Annual Precipitation × Temperature | 0.04884 |  |

**Figure S1.** Saturation Curves for phylogenetic oomycetes (a), BLAST oomycetes (b), fungal pathogens (c), and saprotrophic fungi (d). The bands represent a 95% confidence interval.

**Figure S2.** Venn diagrams for oomycetes (a), fungal pathogens (b) and saprotrophic fungi (c). Venn diagrams showed considerable overlap with respect to fungal pathogen (46%), oomycete (BLAST; 38%) and saprotrophic fungi (46%) OTUs. These diagrams show that there are more unique OTUs found in remnant sites than in disturbed sites. Blue indicates remnant, while red indicates disturbed sites.

**Figure S3.** Differential OTU abundance between Western and Eastern grasslands phylogenetic oomycetes (a), BLAST oomycetes (b), fungal pathogens (c) and saprotrophic fungi (d) in remnant (i) and disturbed (ii) grasslands. Overall, greater turnover is seen in remnant versus disturbed grasslands in all groups. Only significant OTUs are shown; each bar represents one OTU.

a.

i.ii.

b.

i.

ii.

c.

i.

ii.

d.

iii.

**Figure S4**. Differential OTU abundance between remnant and disturbed grasslands for fungal pathogens (a), phylogenetic oomycetes (a), BLAST oomycetes (b), fungal pathogens (c) and fungal saprotrophs (d) in Western (i) and Eastern (ii) grasslands. Only significant OTUs shown; each bar represents an OTU.

a.

i.

ii.

b.

i.

ii.

c.

i.

ii.

d.

i.

ii.

**Figure S5.** The interaction between precipitation and temperature for saprotrophic fungi in remnant grasslands (p = 0.02), based on PSR (phylogenetic species richness) GLM (Table S**2a**). With higher temperature (+ SD), precipitation predicts an increase in PSR; with lower temperature (- SD), precipitation predicts a decrease in PSR. Points represent the raw data; the trendlines are the predicted probability from the GLM.


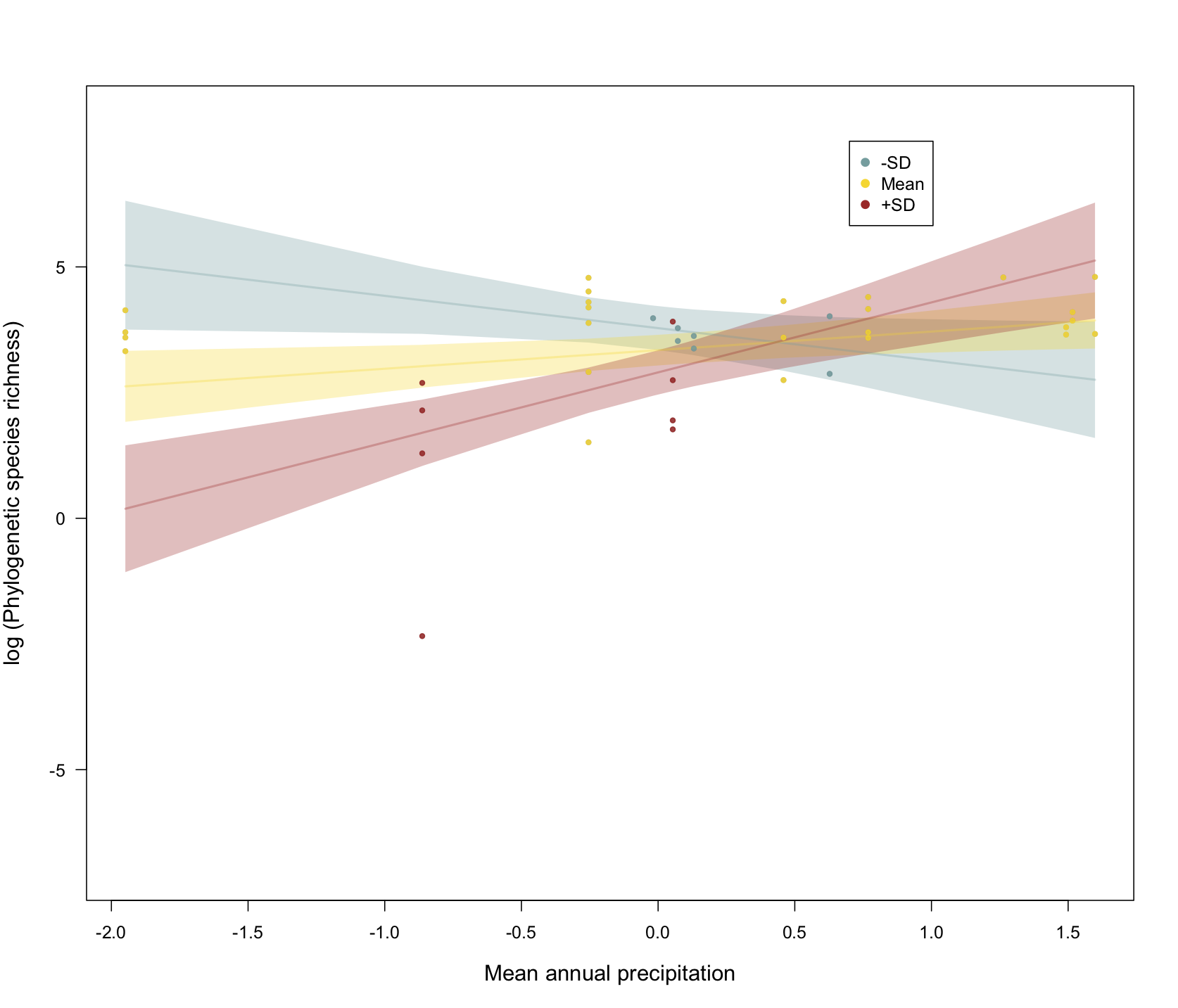

Supplement: Supplementary file 1 — Supplementary Information [file 41396_2020_783_MOESM1_ESM.docx]
